# Disorder-specific grey matter deficits in attention deficit hyperactivity disorder relative to autism spectrum disorder

**DOI:** 10.1017/S0033291714001974

**Published:** 2014-09-17

**Authors:** L. Lim, K. Chantiluke, A. I. Cubillo, A. B. Smith, A. Simmons, M. A. Mehta, K. Rubia

**Affiliations:** 1 Department of Child and Adolescent Psychiatry, Institute of Psychiatry, King's College London, UK; 2 Department of Psychological Medicine, Yong Loo Lin School of Medicine, National University of Singapore, Singapore; 3 Department of Neuroimaging, Institute of Psychiatry, King's College London, UK; 4 NIHR Biomedical Research Centre at South London and Maudsley NHS Foundation Trust (SLaM), London, UK

**Keywords:** ADHD, ASD, cerebellum, magnetic resonance imaging, superior temporal lobe, voxel-based morphometry

## Abstract

**Background.:**

Attention deficit hyperactivity disorder (ADHD) and autism spectrum disorder (ASD) are two common childhood disorders that exhibit genetic and behavioural overlap and have abnormalities in similar brain systems, in particular in frontal and cerebellar regions. This study compared the two neurodevelopmental disorders to investigate shared and disorder-specific structural brain abnormalities.

**Method.:**

Forty-four predominantly medication-naïve male adolescents with ADHD, 19 medication-naïve male adolescents with ASD and 33 age-matched healthy male controls were scanned using high-resolution T1-weighted volumetric imaging in a 3-T magnetic resonance imaging (MRI) scanner. Voxel-based morphometry (VBM) was used to test for group-level differences in structural grey matter (GM) and white matter (WM) volumes.

**Results.:**

There was a significant group difference in the GM of the right posterior cerebellum and left middle/superior temporal gyrus (MTG/STG). *Post-hoc* analyses revealed that this was due to ADHD boys having a significantly smaller right posterior cerebellar GM volume compared to healthy controls and ASD boys, who did not differ from each other. ASD boys had a larger left MTG/STG GM volume relative to healthy controls and at a more lenient threshold relative to ADHD boys.

**Conclusions.:**

The study shows for the first time that the GM reduction in the cerebellum in ADHD is disorder specific relative to ASD whereas GM enlargement in the MTG/STG in ASD may be disorder specific relative to ADHD. This study is a first step towards elucidating disorder-specific structural biomarkers for these two related childhood disorders.

## Introduction

Attention deficit hyperactivity disorder (ADHD) is one of the most commonly diagnosed childhood disorders, defined by age-inappropriate problems with inattention, impulsivity and hyperactivity (APA, 2013). Autism spectrum disorder (ASD) is characterized by abnormalities in social interaction, communication and stereotyped/repetitive behaviours. Both disorders are highly heritable and share high comorbidity (Simonoff *et al.* 2008; Rommelse *et al.* 2010). About 20–50% of ADHD children meet criteria for ASD and 30–80% of ASD children meet criteria for ADHD (Rommelse *et al.* 2010). Apart from comorbidities, ASD patients show some ADHD-typical behaviours such as attention deficits, impulsivity or hyperactivity (Schatz *et al.* 2002) whereas ADHD patients also show some social interaction and communication difficulties, albeit to a smaller degree than ASD patients (Geurts *et al.* 2004).

Despite the reported genetic and behavioural overlap between the two disorders, a diagnosis of ADHD according to DSM-IV (APA, 2000) and ICD-10 (WHO, 1994) was precluded if the symptoms were better accounted for by autism. It has been debated whether the phenotypically similar ADHD-related deficits in ASD are secondary to ASD or a phenocopy, which had prevented the co-diagnosis in DSM-IV and ICD-10 (APA, 2000), or whether they reflect true comorbidity, as suggested in the allowance for co-diagnosis in the current DSM-5 criteria (APA, 2013). The identification of both overlapping and disorder-specific objective neurobiological biomarkers should help to determine to what extent the two disorders differ in their underlying neurobiology.

ADHD is a multi-systemic neurodevelopmental disorder that has consistently been associated with abnormalities in structure, function and inter-regional connectivity of fronto-striato-parieto-temporal and fronto-cerebellar networks (Valera *et al.* 2007; Nakao *et al.* 2011; Rubia, 2011; Cubillo *et al.* 2012*a*; Rubia *et al.* 2014). Structural magnetic resonance imaging (sMRI) studies using region of interest (ROI) analyses have reported reduced grey matter (GM) volume and cortical thickness, most prominently in the cerebellar hemispheres (Castellanos *et al.* 2002; Durston *et al.* 2004; Mackie *et al.* 2007; Valera *et al.* 2007) and cerebellar vermis (Mackie *et al.* 2007; Valera *et al.* 2007), but also in the basal ganglia and frontal regions (Shaw *et al.* 2007). Whole-brain sMRI studies, however, found that the most consistent GM reductions were in the basal ganglia (Nakao *et al.* 2011; Frodl & Skokauskas, 2012).

sMRI studies have reported abnormal GM volumes and cortical thickness in ASD patients relative to controls in several brain regions involved in social, language and executive functions, including prefrontal, temporo-parietal, striatal, limbic and cerebellar regions (Amaral *et al.* 2008; Nickl-Jockschat *et al.* 2012). However, findings have been inconsistent with respect to the direction of GM differences; some studies found increases in GM volumes or cortical thickness (Bonilha *et al.* 2008; Hyde *et al.* 2010) whereas others found decreases (Brun *et al.* 2009; Webb *et al.* 2009; Toal *et al.* 2010) or no differences (Hazlett *et al.* 2005; Scott *et al.* 2009). Studies on the developmental course of brain abnormalities in autism indicate a putative period of abnormal precocious brain growth that is time delimited to the first 2–4 years of life but then plateaus by adolescence and adulthood (Amaral *et al.* 2008; Courchesne *et al.* 2011), with some studies finding arrested growth after adolescence (Amaral *et al.* 2008).

Despite evidence for high comorbidity rates and abnormalities in similar brain systems, in particular in frontal and cerebellar regions, few studies have compared the two disorders to elucidate shared and disorder-specific underlying neurobiological biomarkers. The only sMRI study to date that compared relatively small numbers of 15 ADHD and 15 ASD children found shared reductions in the GM of temporo-parietal regions and also increased GM of the supramarginal gyrus in ASD relative to controls, but not ADHD (Brieber *et al.* 2007). However, the findings did not survive correction for multiple testing. In addition, most ADHD patients were on chronic stimulant medication and two ASD patients took neuroleptic medication that could have confounded the findings, given that long-term psychotropic medication is associated with more normal brain structure (Shaw *et al.* 2009; Murphy, 2010; Nakao *et al.* 2011; Rubia *et al.* 2013*a*). The very few published functional MRI (fMRI) comparisons between the disorders found task-dependent shared and disorder-specific deficits: shared dorsolateral prefrontal deficits during working memory (Chantiluke *et al.* in press *a*); shared dorsolateral prefronto-striato-parietal underactivation and reduced deactivation of posterior cingulate/precuneus default mode regions, but ASD-specific cerebellar overactivation during sustained attention (Christakou *et al.* 2013); ASD-specific underactivation in the ventromedial prefrontal cortex during reversal learning (Chantiluke *et al.* 2014*a*) and ADHD-specific orbitofrontal–striatal underactivation and ASD-specific left frontal overactivation during motor inhibition (Chantiluke *et al.* in press *b*). During temporal discounting, comorbid ADHD and ASD patients had unique brain–behaviour correlation abnormalities relative to controls in ventromedial and lateral frontolimbic regions, followed by the ASD group who had disorder-specific brain–behaviour correlation abnormalities in inferior frontotemporal regions (Chantiluke *et al.* 2014*b*). Finally, a recent resting-state fMRI study reported shared network centrality abnormalities in the precuneus, ADHD-specific increases in network centrality in the right striatum/pallidum and ASD-specific increases in network centrality in predominantly left temporolimbic areas (Di Martino *et al.* 2013).

Given the importance of establishing disorder-specific biomarkers in these two related disorders and evidence of the impact of long-term neurotropic medication on brain structure (Shaw *et al.* 2009; Murphy, 2010; Nakao *et al.* 2011; Rubia *et al.* 2013*a*), we investigated shared and disorder-specific GM and white matter (WM) abnormalities in 44 predominantly medication-naïve ADHD boys, 19 medication-naive ASD boys and 33 healthy boys.

## Method

### Participants

Forty-four mostly medication-naïve right-handed male adolescents with a clinical diagnosis of inattentive/hyperactive-impulsive combined type ADHD, but not ASD, were recruited from out-patient clinics at the South London and Maudsley National Health Service (NHS) Foundation Trust. Diagnosis was assessed by a child psychiatrist using the standardized Maudsley Diagnostic Interview (MDI; Goldberg & Murray, 2002), which assesses ADHD according to DSM-IV-TR criteria (APA, 2000). All patients scored above the clinical cut-off for hyperactive-impulsive/inattentive symptoms on the parental Strengths and Difficulties Questionnaire (SDQ; Goodman, 1997) and the Conners’ Parent Rating Scale (CPRS; Conners *et al.* 1998). ADHD patients were excluded if they scored above the clinical cut-off on the Social Communication Questionnaire (SCQ; Rutter *et al.* 2003). Most ADHD patients (81.8%) were medication naïve, except for six patients (13.6%) who received methylphenidate but had a wash-out of 48 h before scanning and two patients who had been treated with methylphenidate in the past. Nineteen right-handed medication-naïve male adolescents with a diagnosis of ASD, but not ADHD, were recruited through out-patient clinics. The ASD diagnosis was made using ICD-10 research diagnostic criteria (WHO, 1994), confirmed by the Autism Diagnostic Interview – Revised (ADI-R; Lord *et al.* 1994) and the Autism Diagnostic Observation Schedule (ADOS; Lord *et al.* 1989). ASD patients were excluded if they scored above 7 on the Hyperactivity/Inattention ratings on the SDQ. Five boys had high functioning autism (HFA) and 14 boys had Asperger's disorder. ADOS modules were selected based on verbal ability and age (Lord *et al.* 2000). Because of the high (verbal) functioning of the ASD boys, ADOS module 4 was used. Thirteen ASD boys reached the SCQ score cut-off. However, the algorithm used to obtain this score is not entirely indicative of clinical impairment and algorithms incorporating restricted and repetitive behaviours are more sensitive, as evidenced by the use of an algorithm that includes stereotyped behaviours in the new ADOS-2 (Lord *et al*. 2012). The ADI-R and ADOS are used in conjunction to obtain a holistic view and reliable diagnosis of an individual (Papanikolaou *et al.* 2009). All ASD participants scored above the clinical cut-off on the social, communication and restrictive and repetitive behaviour domains of the ADI-R and this, alongside the ADOS scores, was used to ensure that each adolescent met the criteria for ASD.

Thirty-three, age-matched, right-handed healthy boys were recruited through advertisement and scored below clinical thresholds on the SDQ and SCQ (Table 1). Participants were excluded if they had comorbid psychiatric disorders as assessed by the MDI, including learning disabilities, reading, speech or language disorder, neurological abnormalities, epilepsy, substance abuse and an IQ < 70 on the Wechsler Abbreviated Scale of Intelligence (WASI; Wechsler, 1999). Participants were reimbursed £50 for taking part in the study and written informed consent was obtained. The study was approved by the Camberwell St Giles Research Ethics Committee.

**Table 1.** Sample characteristics of participants

Variables	CON (33)	ADHD (44)	ASD (19)	*F* test	*p* value^a^	Subject contrast
Age (years)	14.3 (2.52)	13.6 (1.87)	14.9 (1.86)	2.68	n.s.	–
IQ	110 (11.5)	92.2 (11.7)	113 (15.7)	27.3	*<* 0.001	ADHD<ASD, CON
SDQ hyperactive/inattentive	1.97 (1.77)	8.49 (1.94)	4.74 (1.91)	107.4	*<* 0.001	ADHD>ASD>CON
SDQ emotional distress	1.09 (1.53)	3.72 (2.82)	4.26 (2.88)	13.8	*<* 0.001	ASD, ADHD>CON
SDQ conduct subscale	0.72 (1.49)	5.67 (2.42)	2.16 (1.95)	55.0	*<* 0.001	ADHD>ASD>CON
SDQ peer relationships	0.88 (1.31)	3.77 (2.37)	6.21 (2.30)	43.0	*<* 0.001	ASD>ADHD>CON
SDQ prosocial behaviour	8.56 (2.17)	5.97 (2.12)	5.16 (2.22)	19.0	*<* 0.001	CON>ASD, ADHD
SDQ total scores	4.66 (4.53)	21.6 (5.88)	17.4 (5.46)	91.7	*<* 0.001	ADHD>ASD>CON
SCQ total score	1.96 (2.37)	8.72 (4.10)	23.3 (5.39)	155.8	*<* 0.001	ASD>ADHD>CON
CPRS-R total *T* score	44.8 (5.88)	76.1 (7.54)	58.5 (6.50)	151.4	*<* 0.001	ADHD>ASD>CON
ADOS social scores	–	–	7.35 (3.92)			
ADOS communication scores	–	–	2.24 (1.48)			
ADOS communication and social scores	–	–	9.59 (5.03)			
ADOS stereotyped behaviour scores	–	–	1.12 (0.99)			
ADI-R social scores	–	–	16.3 (4.59)			
ADI-R communication scores	–	–	13.9 (3.77)			
ADI-R repetitive behaviour scores	–	–	5.17 (2.85)			

IQ, Intelligence quotient as assessed with the Wechsler Abbreviated Scale of Intelligence (WASI); ADHD, attention deficit hyperactivity disorder; ASD, autism spectrum disorder; CON, controls; CPRS-R, revised Conners’ Parent Rating Scale; SDQ, Strengths and Difficulties Questionnaire; SCQ, Social Communication Questionnaire; ADI-R, Autism Diagnostic Interview – Revised; ADOS, Autism Diagnostic Observation Schedule; n.s., not significant.

Values given as mean (standard deviation).

a Bonferroni correction.

### MRI image acquisition

Images were acquired using a 3-T GE Signa HDx system (General Electric, USA) at the Centre for Neuroimaging Sciences, Institute of Psychiatry, King's College London, UK. The body coil was used for radio frequency (RF) transmission and an eight-channel head coil for RF reception. High-resolution structural three-dimensional (3D) T1-weighted magnetization-prepared rapid gradient-echo (MPRAGE) images were acquired. Full brain and skull coverage was required for each subject and detailed quality control was carried out on all MR images according to previously published quality control criteria (Simmons *et al.* 2011).

### VBM-DARTEL image preprocessing

The images were first visually inspected for artefacts and structural abnormalities. Next, a VBM analysis (Ashburner & Friston, 2000) was conducted to investigate group differences in GM volumes using SPM8 software (Statistical Parametric Mapping, Wellcome Department of Imaging Neuroscience, London, UK). The T1-weighted volumetric images were preprocessed using the VBM protocol with modulation (Ashburner, 2007), where the images were first segmented into GM, WM and cerebrospinal fluid (CSF). The DARTEL algorithm was applied to the segmented brain tissues to generate a study-specific template and to achieve an accurate inter-subject registration with improved realignment of smaller inner structures (Yassa & Stark, 2009). The normalized modulated segmented GM/WM images were next affine transformed into Montreal Neurological Institute (MNI) space and smoothed with an isotropic Gaussian kernel of 8 mm at full-width half-maximum, providing a balance between predicted subcortical and cortical effects, and to accommodate the assumptions of Gaussian random field theory and the matched filter theorem.

### VBM analysis

Group differences were evaluated for GM/WM volumes obtained in the tissue segmentation step of the VBM-DARTEL preprocessing. The total brain volume (TBV) was calculated as the sum of GM and WM volumes. The normalized modulated and smoothed GM/WM images in each group were entered into voxel-wise ANOVAs using SPM8. We used a cluster-defining voxelwise threshold of *p* < 0.01 (uncorrected) and a stringent cluster threshold of *p* < 0.05 family-wise error (FWE) rate corrected for all the analyses. Cluster sizes were adjusted for smoothness non-uniformity by means of the VBM5.1 toolbox (Hayasaka *et al.* 2004). To test for correlations between structural abnormalities in GM and clinical symptoms, simple regression analyses were performed within each group.

## Results

### Participant characteristics

Groups did not differ significantly in age but there were significant differences in IQ (*F*_2,93_ = 27.3, *p* < 0.001) (Table 1). *Post-hoc* analyses showed that this was because the ADHD boys had lower IQs than healthy and ASD boys (*p* < 0.01), which is typical of the ADHD population (Kuntsi *et al.* 2004). When a covariate differs between groups because it is associated with a particular condition, and groups have not been selected randomly, an ANCOVA covarying for IQ to adjust for this variable would be inappropriate, as it would violate the basic ANCOVA assumption that the covariate is independent of the selected groups (Miller & Chapman, 2001; Dennis *et al.* 2009). However, in our study, to assess the potential effect of IQ on GM group differences, GM volumes were correlated with IQ within each group. As expected, based on the selection criteria, group differences were significant in CPRS, SDQ hyperactivity and SCQ scores (Table 1).

### VBM-DARTEL analysis of GM/WM volume differences

There was a significant group difference in total GM volume and TBV (Table 2). *Post-hoc* analyses showed that ADHD boys had significantly smaller total GM volume and TBV compared to the other two groups. Hence, TBV was entered as a covariate in the subsequent analyses.

**Table 2.** Group differences between adolescents with ADHD, ASD and healthy controls in global brain volume

	CON (*n* = 33)	ADHD (*n* = 44)	ASD (*n* = 19)	*F* test	*p* value^a^	Subject contrast
GM volume (ml)	783 (54.3)	747 (57.6)	792 (55.0)	5.92	0.004	ASD, CON>ADHD
WM volume (ml)	512 (43.1)	494 (45.0)	516 (33.5)	2.43	0.094	–
TBV (ml)	1295 (95.8)	1242 (100.0)	1307 (86.6)	4.41	0.015	ASD, CON>ADHD

ADHD, Attention deficit hyperactivity disorder; ASD, autism spectrum disorder; CON, controls; GM, grey matter; WM, white matter; TBV, total brain volume ( = GM volume + WM volumes).

Values given as mean (standard deviation).

a Bonferroni correction.

**Fig. 1.**
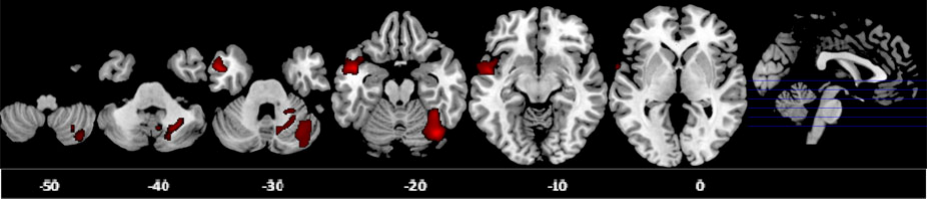
Axial sections of grey matter (GM) reduction in the right posterior cerebellum in attention deficit hyperactivity disorder (ADHD) patients compared with controls and autism spectrum disorder (ASD) patients; and GM enlargement in the left middle/superior temporal gyrus in ASD patients relative to controls as revealed by the *F* test (*p* < 0.05), family-wise error (FWE) corrected at the cluster level. Axial slices are marked with the *z* coordinate as distance in millimetres from the anterior–posterior commissure. The right side of the image corresponds to the right side of the brain.

Voxel-wise ANCOVA (*p* < 0.05 FWE-corrected) showed a significant group effect in the GM volumes of the right posterior cerebellum [effect size (EF) = 0.40, *R*^2^ = 0.29] and left middle/superior temporal gyrus (MTG/STG: EF = 0.17, *R*^2^ = 0.14). *Post-hoc* analyses showed that ADHD boys had significantly smaller right posterior cerebellar GM compared to the other two groups, which did not differ from each other. ASD boys had significantly larger GM in the left MTG/STG compared with controls. At a more lenient cluster threshold of *p* < 0.05 uncorrected, this was also significant relative to ADHD boys; in addition, ASD boys had a larger left medial frontal GM volume compared to both groups (Table 3, Fig. 1).

**Table 3.** Group differences in GM volumes between adolescents with ADHD, ASD and healthy controls

Brain region	BA	Peak MNI coordinates (x, y, z)	Number of voxels	*p* value of cluster (FWE corrected)	Subject contrast
Right posterior cerebellum	–	39, −67, −20	2761	0.01	ASD, CON>ADHD
Left middle/superior temporal gyrus	21/38	−50, 3, −17	1956	0.04	ASD>CON, ADHD^a^
Left medial frontal gyrus	6	−12, −9, 57	516	0.049*	ASD>CON^a^, ADHD^a^

GM, Grey matter; ADHD, attention deficit hyperactivity disorder; ASD, autism spectrum disorder; BA, Brodmann area; MNI, Montreal Neurological Institute; CON, controls; FWE, family-wise error.

a At a lenient significance threshold of cluster *p* < 0.05 uncorrected.

Given that one of the most significant sMRI findings in ADHD is that of reduced GM volumes in the basal ganglia (Nakao *et al.* 2011; Frodl & Skokauskas, 2012), we conducted an additional ROI analysis extracting data for the bilateral basal ganglia using MARSBAR (Brett *et al.* 2002); no significant group differences were observed. The simple regression analyses between GM volumes and IQ and clinical ratings within each group revealed no significant correlation. No significant group differences were observed in WM volume.

Given that long-term stimulant medication is associated with more normal brain structure (Shaw *et al.* 2009; Murphy, 2010; Nakao *et al.* 2011; Rubia *et al.* 2013*a*), including the cerebellum (Bledsoe *et al.* 2009), we tested whether the cerebellum differences survived when we only compared the 36 medication-naïve ADHD boys with the other two groups. The findings remained unchanged.

## Discussion

To our knowledge, this is the second sMRI study comparing non-comorbid ADHD boys and non-comorbid ASD boys, in a relatively larger sample than the previous study (Brieber *et al.* 2007). Moreover, we included mostly medication-naïve participants and used a more stringent significance threshold corrected for multiple comparisons. The key finding is that non-comorbid, mostly medication-naïve, ADHD boys had a disorder-specific reduction in the right posterior cerebellar GM relative to non-comorbid medication-naïve ASD boys, suggesting that this may be a disorder-specific biomarker to differentiate between these two neurodevelopmental disorders. Furthermore, the finding survived when we only included the 36 medication-naïve ADHD boys in the analysis, thus excluding potential confounds of long-term stimulant medication treatment. ASD boys, by contrast, showed a GM enlargement in the left MTG/STG relative to controls, which was disorder specific relative to ADHD at a more lenient threshold.

The finding of an ADHD-specific GM deficit in the right posterior cerebellum extends prior literature on consistent deficits in ADHD in this region by showing for the first time that this is disorder specific relative to ASD. Reduction in cerebellar hemispheric volumes is one of the most consistent findings of sMRI studies in ADHD (Durston *et al.* 2004; Biederman *et al.* 2008; Montes *et al.* 2011; de Zeeuw *et al.* 2012; Lim *et al.* 2013), with the largest effect size in a meta-analysis of ROI sMRI studies (Valera *et al.* 2007). Reduced cerebellar volumes are also observed in longitudinal studies, where the deficit is sustained throughout adolescence (Castellanos *et al.* 2002; Mackie *et al.* 2007) and adulthood (Proal *et al.* 2011).

The cerebellum is one of the few brain regions that have been associated directly with ADHD diagnostic status and clinical outcome. Thus, ADHD patients with worse clinical outcome showed a progressively smaller total cerebellar volume with age, attributable mainly to the deviant trajectory of the inferior–posterior hemispheres, relative to healthy controls (Mackie *et al.* 2007). Furthermore, reduced (right) cerebellar volumes have been shown to be specifically associated with diagnostic status, rather than to be an endophenotype of ADHD, as deficits were not observed in unaffected siblings (Durston *et al.* 2004).

The cerebellum is one of the latest brain structures to fully develop. In particular, the cerebellar hemispheres reach their peak volume as late as around age 18 years, and the structural development of the different cerebellar regions parallels those prefrontal regions they are connected with to form the late-developing fronto-cerebellar networks that mediate higher-level motor, cognitive and affective functions (Mackie *et al.* 2007; Tiemeier *et al.* 2010; Arnsten & Rubia, 2012). It is therefore plausible that our finding of a disorder-specific reduction in the right lateral cerebellar GM in ADHD boys relative to ASD boys, together with prior consistent evidence of smaller cerebellar hemisphere volumes in ADHD boys (Castellanos *et al.* 2002; Durston *et al.* 2004; Mackie *et al.* 2007; Valera *et al.* 2007; Biederman *et al.* 2008; Montes *et al.* 2011; Proal *et al.* 2011; de Zeeuw *et al.* 2012; Lim *et al.* 2013) could potentially reflect a maturational delay in ADHD. This would parallel the delay in GM thickness development of prefrontal and temporoparietal regions that co-develop with the cerebellum (Shaw *et al.* 2007, 2012).

The cerebellum has traditionally been considered to be primarily involved in motor control. However, lesion and fMRI studies have consistently demonstrated its involvement in a wide range of cognitive and affective functions, in particular sustained and shifting attention (Schmahmann, 2004), working memory (Ravizza *et al.* 2006), inhibitory control (Rubia *et al.* 2007, 2013*b*), temporal information processing (Rubia & Smith, 2004; Rubia, 2006; Wiener *et al.* 2010; Noreika *et al.* 2012) and emotion regulation (Allen *et al.* 1997). This is further underscored by the extensive connections of the lateral cerebellar hemispheres to the prefrontal cortex and the basal ganglia, forming fronto-striato-cerebellar networks (Arnsten & Rubia, 2012). Based on lesion studies (Exner *et al.* 2004) and meta-analyses (Stoodley & Schmahmann, 2009), the anterior part of the cerebellum is particularly involved in motor and sensory functions, the medial part in emotion processes and the lateral posterior region, found to be abnormal in ADHD in this study, in higher-level cognitive abilities such as attention (Kellermann *et al.* 2012; Li *et al.* 2012), inhibition (Rubia *et al.* 2007, 2013*b*), working memory (Stoodley & Schmahmann, 2010; Massat *et al.* 2012; Stoodley *et al.* 2012) and timing functions (O'Reilly *et al.* 2008; Wiener *et al.* 2010).

The right-hemispheric location of the cerebellar GM deficit finding in ADHD is also important, given that the right posterior cerebellar hemisphere has been found to be particularly relevant for attention and working memory (Kellermann *et al.* 2012; Li *et al.* 2012; Bernard & Seidler, 2013). ADHD children have consistent deficits in these above-mentioned cognitive functions, especially working memory, sustained attention (Willcutt *et al.* 2005; Rubia, 2011; Cubillo *et al.* 2012*a*), inhibition (Lijffijt *et al.* 2005) and timing functions (Rubia *et al.* 2009*a*; Noreika *et al.* 2012). In ASD, impairments in these functions are more controversial, with many negative findings with respect to selective and sustained attention (Johnson *et al.* 2007; Rommelse *et al.* 2011) and working memory (Rommelse *et al.* 2011), and less consistent evidence for inhibition (Rommelse *et al.* 2011) and timing impairment (Falter *et al.* 2012). Furthermore, when ADHD comorbidity is excluded and compared to ADHD, ASD patients are less impaired in these cognitive functions (Johnson *et al.* 2007; Rommelse *et al.* 2011).

Evidence for cerebellar GM abnormalities in ADHD are further supported by diffusion tensor imaging (DTI) studies that have reported reduced fractional anisotropy (FA) in the WM tracts of the right middle (Bechtel *et al.* 2009; Kobel *et al.* 2010; Chuang *et al.* 2013) and left inferior cerebellar peduncle (Nagel *et al.* 2011) in ADHD patients compared to controls, suggesting deficient structural connectivity between the cerebellum and prefrontal regions.

Cerebellar GM deficits in ADHD also echo evidence for abnormal function of this region based on fMRI studies that have found the lateral and medial cerebellum to be abnormal in their activation in ADHD patients together with frontostriatal deficits, most consistently during tasks of sustained and selective attention (Rubia *et al.* 2009*b*; Cubillo *et al.* 2012*a*), timing (Rubia *et al.* 2009*a*; Valera *et al.* 2010; Vloet *et al.* 2010; Hart *et al.* 2012) and inhibition (Rubia *et al.* 2011, 2013*a*; Cubillo *et al.* 2012*b*; Hart *et al.* 2014).

Similarly, fMRI studies have detected abnormal functional connectivity between the cerebellum and prefrontal, striatal and parietal regions in ADHD patients during attention, timing (Rubia *et al.* 2009*b*; Vloet *et al.* 2010) and working memory (Massat *et al.* 2012) performance, suggesting that different task-relevant fronto-striato-cerebellar networks are dysfunctional in ADHD.

The disorder specificity of the right posterior cerebellar GM deficit relative to ASD is intriguing. Although cerebellar abnormalities are consistent findings in sMRI studies of ASD, there is debate regarding the nature and consistency of these cerebellar alterations. Some studies have found the cerebellum to be enlarged (Palmen *et al.* 2005; Bonilha *et al.* 2008), smaller (Webb *et al.* 2009; Toal *et al.* 2010) or not to differ compared with controls (Hazlett *et al.* 2005; Scott *et al.* 2009). The age of ASD patients is likely to play an important role given that, in early infancy and childhood, ASD is associated with significantly enlarged GM volumes relative to controls; later on, in adolescence and adulthood, there is evidence for arrested growth relative to controls (Amaral *et al.* 2008). By adolescence, some studies found normal TBV (Hazlett *et al.* 2005; Scott *et al.* 2009), suggesting that the precocious overgrowth from the first years of life may normalize with age by adolescence. However, most of this evidence is based on cross-sectional data, and longitudinal data are needed to elucidate developmental growth trajectories. Our findings, however, suggest that, by adolescence, the cerebellar hemispheres are disorder-specifically reduced in ADHD relative to ASD who have normal TBV and cerebellar GM at this age point.

Importantly, we found that the ADHD-specific GM deficit relative to the other two groups in the right posterior cerebellum survived when we only included medication-naïve patients. Medication naivety is crucial for neuroimaging studies, as we have shown in a meta-regression analysis that long-term stimulant medication is associated with more normal brain structure in the basal ganglia in ADHD (Nakao *et al.* 2011). Retrospective analyses have found that medication-naïve ADHD patients have more abnormal GM than long-term medicated ADHD patients in the cerebellum (Bledsoe *et al.* 2009) and other ADHD-relevant areas (Shaw *et al.* 2009; Ivanov *et al.* 2010; Rubia *et al.* 2013*a*). Therefore, long-term stimulant medication is not a confound in our results.

ASD boys also had larger left MTG/STG GM volumes relative to controls, and at a more lenient threshold relative to ADHD boys. The STG is involved in auditory processing including language and has been implicated in social cognition (Pelphrey *et al.* 2004). Language deficits are a core feature of ASD and failure to develop normal language comprehension is an early warning sign of autism (Eyler *et al.* 2012). Several studies have reported larger left temporal GM volume in ASD patients relative to healthy controls (Hazlett *et al.* 2006; Rojas *et al.* 2006; Knaus *et al.* 2009; Verhoeven *et al.* 2010; Cauda *et al.* 2011), which was also correlated with social and communication deficits (Rojas *et al.* 2006; Verhoeven *et al.* 2010). In addition, one study has shown that ASD patients, compared to healthy controls, did not show the normal age-related reductions in MTG/STG cortical volume and thickness during adolescence and adulthood, suggesting cortical dysmaturation in a brain region that is crucial to social cognition and language (Raznahan *et al.* 2010). Several fMRI studies have suggested that abnormal activation in the left MTG/STG [especially Brodmann area (BA) 21, as found in this study] may play a central role in the typical language impairment in ASD (Redcay & Courchesne, 2008; Eyler *et al.* 2012). In particular, a failure of the left temporal cortex to specialize for language during early development may reflect a fundamental early neural developmental pathology in autism (Eyler *et al.* 2012). Furthermore, a DTI study reported abnormalities in the microstructural organization of the WM tracts involving the STG and temporal stem in autistic patients compared to controls (Lee *et al.* 2007). Our finding of a larger STG GM volume in ASD parallels the findings of Brieber *et al*. (2007), who reported an increased GM volume in the adjacent right supramarginal gyrus in ASD relative to controls and ADHD, although their findings did not survive correction for multiple comparisons. In addition, our finding of abnormalities in the left temporal gyrus in ASD patients parallels the findings of Di Martino *et al*. (2013), who found disorder-specific functional network abnormalities in ASD relative to ADHD and controls in limbic networks, including the left planum temporale and temporal cortex. The disorder specificity of this abnormality may only have been detected at a more lenient threshold because of the relatively smaller numbers in the ASD group; replications in larger samples are therefore needed to corroborate these findings. The relatively small numbers of participants may also have prevented us from finding a structural deficit in the basal ganglia in ADHD, which was observed in two meta-analyses of sMRI studies (Nakao *et al.* 2011; Frodl & Skokauskas, 2012).

We found no WM differences between groups. sMRI findings have been inconclusive, with some studies finding WM abnormalities in ADHD (Castellanos *et al.* 2002; Durston *et al.* 2004) and ASD (McAlonan *et al.* 2005; Bonilha *et al.* 2008), but not others (Palmen *et al.* 2005; Brun *et al.* 2009; Batty *et al.* 2010). The sample sizes, especially for the ASD group, may have been too small to detect any WM abnormalities. Moreover, changes in WM integrity may be assessed more accurately using DTI (Whitwell, 2009).

A strength of this study is that all ASD and most ADHD patients were medication naïve. Furthermore, our findings survived a subanalysis in only medication-naïve ADHD patients. This is important because stimulant medication, the gold standard medication for ADHD, and selective serotonin reuptake inhibitors (SSRIs), which are sometimes used in ASD, have been associated with differences in brain structure including the cerebellum (Bledsoe *et al.* 2009; Ivanov *et al.* 2010; Murphy, 2010; Nakao *et al.* 2011; Rubia *et al.* 2013*a*). Another strength is the careful diagnosis of ASD patients who were non-comorbid with ADHD, using the ICD-10, ADI and ADOS, and of ADHD patients who were non-comorbid with ASD, using the CPRS, SDQ and SCQ, both without other comorbid psychiatric diagnoses. Although the inclusion of only males enhances the homogeneity of the group and is based on the higher prevalence in boys for both disorders (Rommelse *et al.* 2010), it limits the generalizability to females with the disorders. Furthermore, we included only high-functioning adolescents with ASD and Asperger's disorder, and the combined subtype of ADHD, which limits the generalizability to other subtypes within ASD or ADHD. Additionally, although in this study we carefully excluded comorbidity between the two disorders, future studies should elucidate to what extent the comorbid conditions share the same deficits observed in non-comorbid ADHD and non-comorbid ASD or whether they are different in their neurobiological substrates. Another limitation is the relatively smaller sample size of the ASD group, although it was still somewhat larger than that of the only other study that compared brain structure between these two disorders (Brieber *et al.* 2007). Finally, in view of evidence for increased testosterone levels in ASD during puberty (Geier & Geier, 2007), future studies should include measures of pubertal status and hormonal measures.

In summary, using a stringent threshold corrected for multiple comparisons and including mostly medication-naïve, carefully diagnosed non-comorbid groups of ADHD and ASD boys, we found that ADHD boys had a disorder-specific GM volume reduction in the right posterior cerebellum whereas ASD boys had a disorder-specific GM volume enlargement in the left MTG/STG, albeit at a more lenient significance level. The findings represent a first step towards the delineation of disorder-specific structural biomarkers for these two related disorders.
